# A comparative study of collagen morphology and joint strength in anterior cruciate ligament repair and reconstruction models in rabbits

**DOI:** 10.14202/vetworld.2025.1313-1321

**Published:** 2025-05-25

**Authors:** Andre Yanuar, Andi Isra Mahyuddin, Nucki Nursjamsi Hidajat, Hasrayati Agustina, Nicolaas Cyrillus Budhiparama, Nur Atik

**Affiliations:** 1 Doctoral Program, Faculty of Medicine, Universitas Padjadjaran, Bandung, West Java, Indonesia; 2 Department of Orthopaedic and Traumatology, Santo Borromeus Hospital, Bandung, West Java, Indonesia; 3 Faculty of Mechanical and Aerospace Engineering, Institut Teknologi Bandung (ITB), Bandung, West Java, Indonesia; 4 Department of Orthopaedic and Traumatology, Faculty of Medicine, Universitas Padjadjaran/Dr. Hasan Sadikin General Hospital, Bandung, West Java, Indonesia; 5 Department of Pathology Anatomy, Faculty of Medicine, Universitas Padjadjaran/Dr. Hasan Sadikin General Hospital, Bandung, West Java, Indonesia; 6 Department of Orthopaedic and Traumatology, Faculty of Medicine, Universitas Airlangga, Surabaya, East Java, Indonesia; 7 Department of Orthopaedics, Leiden University Medical Centre, Leiden, Netherlands; 8 Department of Biomedical Sciences, Faculty of Medicine, Universitas Padjadjaran, Bandung, West Java, Indonesia

**Keywords:** anterior cruciate ligament repair, biomechanics, osteointegration, rabbit model, type I collagen

## Abstract

**Background and Aim:**

Anterior cruciate ligament (ACL) repair offers several theoretical advantages over reconstruction, including preservation of native proprioception and reduced donor-site morbidity. However, the current experimental models are predominantly limited to ACL reconstruction, leaving a critical gap in ACL repair research. This study introduces a novel rabbit model to evaluate osteointegration and mechanical strength at the tendon/ligament-bone interface following ACL repair and reconstruction.

**Materials and Methods:**

Six male New Zealand White rabbits (*Oryctolagus cuniculus*), aged 90 ± 0 days and weighing 2.50 ± 0.20 kg, were randomly assigned to two groups: ACL reconstruction (n = 3) using the extensor digitorum longus tendon graft and ACL repair (n = 3) using the Krackow suture technique at the femoral attachment. Specimens were collected 6 weeks postoperatively for histological evaluation of Sharpey’s-like fibers, immunohistochemical analysis of types I and III collagen, and biomechanical tensile testing.

**Results:**

All surgical procedures were completed without complications. Histological analysis showed greater numbers of Sharpey’s-like fibers in the reconstruction group (6.33 ± 0.58%) compared to the repair group (5.67 ± 1.6%), though not statistically significant (p > 0.05). Type I collagen fibers were significantly longer in the reconstruction group in both longitudinal (3.10 ± 0.05 μm vs. 2.97 ± 0.04 μm) and transverse (1.94 ± 0.09 μm vs. 1.81 ± 0.05 μm) dimensions (p < 0.05). Type III collagen dimensions did not differ significantly. The mean tensile failure load was higher in the reconstruction group (105.96 ± 63.37 N) than in the repair group (62.56 ± 20.11 N), though this difference was not statistically significant (p > 0.05).

**Conclusion:**

This study establishes a reproducible and cost-effective ACL repair model in rabbits and confirms that tendon-bone osteointegration occurs in both ACL repair and reconstruction. Superior biomechanical strength and enhanced type I collagen integration in the reconstruction group underscore current clinical outcomes favoring reconstruction. This model offers a valuable platform for exploring biological augmentation strategies to enhance ACL repair efficacy.

## INTRODUCTION

Knee injuries are among the most prevalent musculoskeletal disorders, with an annual incidence of 48 cases/1000 individuals. Ligament injuries represent approximately 9% of these cases, with anterior cruciate ligament (ACL) injuries being the most frequently encountered type [1]. ACL reconstruction is currently regarded as the standard treatment for unstable ACL injuries; however, its outcomes remain suboptimal. This procedure does not fully restore neuromuscular function [2–4], and approximately 45% of patients who undergo ACL reconstruction develop early-onset osteoarthritis within a decade [5]. Historically, ACL repair was employed before the widespread ado-ption of reconstruction, but due to its unsatisfactory results, reconstruction became the preferred app-roach over repair [6–8]. A meta-analysis encompassing 2,401 patients across 28 studies confirmed that ACL reconstruction demonstrates superior survivorship when compared with ACL repair [9].

Despite this, ACL repair presents several theoretical advantages over reconstruction, such as the preservation of proprioceptive and kinematic function – thereby maintaining neuromuscular integrity – as well as the conservation of bone stock and reduced donor-site morbidity, given that no graft harvesting is necessary [10, 11]. Owing to these potential benefits, ongoing research seeks to refine surgical techniques and explore biological augmentation strategies aimed at improving clinical outcomes in ACL repair [6, 9, 10, 12]. Nevertheless, experimental animal studies have largely focused on ACL reconstruction [13–16].

Performing ACL procedures in animal models remains technically demanding. While larger animals such as dogs, sheep, and goats are frequently utilized due to anatomical and biomechanical similarities to humans, we selected rabbits as our experimental model because of their ease of handling and lower housing and care requirements [13, 14]. Introducing this innovative surgical technique in rabbits offers a cost-effective and manageable model for investigating tendon/ligament-to-bone integration and evaluating cellular behavior, such as migration and survival [13].

Despite the theoretical advantages of ACL repair over reconstruction – such as the preservation of proprioceptive function, reduced donor site morbidity, and avoidance of graft harvesting – the clinical outcomes of ACL repair remain inconsistent and inferior to those of reconstruction. Most current knowledge on tendon-to-bone healing has been derived from ACL reconstruction models, with a lack of well-established and reproducible experimental models specifically designed for ACL repair. In addition, the biomechanical and histological mechanisms underlying the osteointegration process in ACL repair remain poorly characterized. There is a notable paucity of preclinical studies that dire-ctly compare collagen morphology and mechanical properties at the tendon/ligament-bone interface between ACL repair and reconstruction, particularly in cost-effective and manageable small animal models. This gap hinders the advancement of optimized surgical techniques and biological augmentation strategies aimed at improving ACL repair outcomes.

The present study aims to develop and vali-date a novel ACL repair model using New Zealand White rabbits and to compare it with an established ACL reconstruction model. Specifically, this research investigates the extent of osteointegration at the ten-don/ligament-bone interface through quantitative assessment of Sharpey’s-like fibers, collagen type I and III immunoexpression, and biomechanical tensile strength. By establishing a reliable and accessible small animal model for ACL repair, this study seeks to provide foundational data that will support future investigations into the biological and mechanical enhancement of ACL repair techniques.

## MATERIALS AND METHODS

### Ethical approval

All animal procedures were conducted in accor-dance with the guidelines of Padjadjaran University’s Animal Experiment Ethics Committee (No. 380/UN6.KEP/EC/2023). The number of experimental animals was minimized in accordance with the 3R principle – Replacement, Reduction, and Refinement. This study employed a limited number of animals to preliminarily evaluate whether the newly implemented ACL repair technique in rabbits enables successful ligament-to-bone integration. A minimal number of animals were also included in the control group, given the abundance of prior research on ACL reconstruction in rabbits.

### Study period and location

The study was conducted from July 2023 to March 2024 at Animal Laboratory, Faculty of Medicine, Padjadjaran University, Anatomical Pathology Labor-atory at Santo Borromeus Hospital, Bandung, and Biomechanical Laboratory at Bandung Institute of Technology, Bandung, Indonesia.

### Experimental animals

To ensure data validity, the sample size was deter-mined based on statistical power analysis using the Lemeshow formula. The minimum calculated sample size per group was 3.58, which was rounded down to three animals per group due to the pilot nature of this study, which aimed to evaluate the method’s feasibility, detect technical challenges, and observe treatment effects.

Six healthy male New Zealand White rabbits (*Oryctolagus cuniculus*), with a mean age of 90 ± 0 days and a mean body weight of 2.50 ± 0.20 kg, were obtained from the Padjadjaran University Animal Laboratory. These parameters align with the previous study by Bachy *et al*. [13] employing rabbits as a model for ACL reconstruction. Male rabbits were chosen to minimize biological variation related to sex. The animals were randomly allocated into two groups: the reconstruction group (n = 3) and the repair group (n = 3), with surgical procedures performed on both hind limbs.

### Anesthesia and pre-operative preparation

All personnel involved were trained in proper animal handling. All procedures were conducted under the supervision of a licensed veterinarian. General anesthesia was induced through intramuscular administration of ketamine (20 mL/rabbit). Prophylactic antibiotics were administered intramuscularly using cefazolin (20 mg/kg) before skin incision. The anterior surfaces of both knees were shaved, cleaned with an iodine-based alcohol-free antiseptic, and treated with depilatory cream.

### Surgical procedures

#### ACL reconstruction

**Figure 1:**
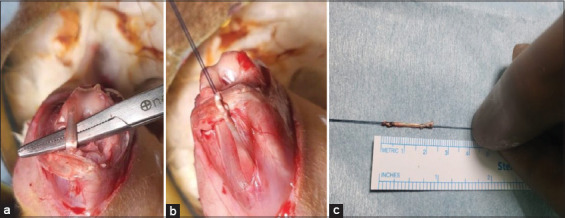
(a) The extensor digitorum longi tendon was identified after arthrotomy. (b)Tendon autograph preparation; suturing of the proximal edge of the tendon graft using the Krackow technique. (c) The tendon graft is 2 cm in length.

**Figure 2:**
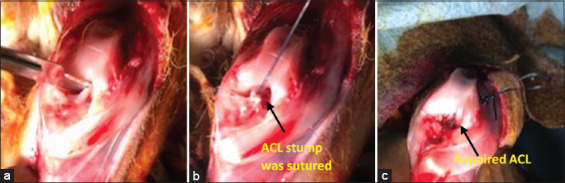
(a) Transection of anterior cruciate ligament attachment in medial site of the lateral femoral condyle. (b) Suture of the ligament edge using the Krackow technique. (c) The suture was passed through the femoral tunnels with the help of needle 23 and knots were made until the size of the knot exceeded the diameter of the bone tunnel.

Under sterile conditions, each rabbit was placed in the supine position, with the knee exposed through a sterile drape. A median incision was made to access the knee, and the extensor digitorum longus tendon was harvested (minimum length: 20 mm; diameter: 1.8 mm) (Figure 1). The harvested autograft was cleaned of muscle tissue and sutured at both ends using the Krackow technique (Figures 2a and b). A medial parapatellar arthrotomy was performed, with the knee slightly flexed and the patella displaced laterally.

**Figure 3:**
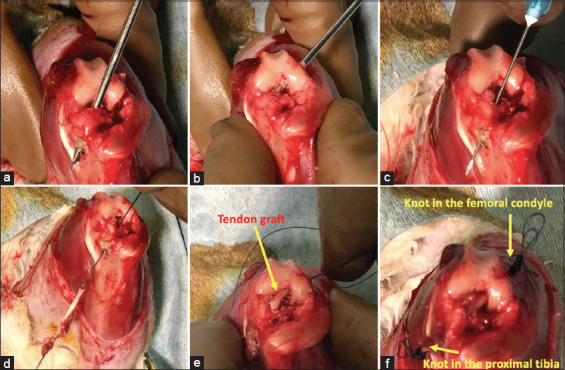
(a) Tunnel preparation; 1.8 mm Kirschner wire was drilled at the anatomical insertions of the anterior cruciate ligament in the tibia and in (b) femur. (c) The tendon graft was passed through the tunnels with the help of needle 23. (d) It started from the tibial tunnel, (e) then continued through the femoral tunnel so that part of the tendon graft enters the tunnel. (f) The tendon graft ends under tension with their sutures, knots were made up to 6 times in the tibial and femoral side until the size of the knot exceeds the diameter of the bone tunnel and resembles an endobutton implant.

The ACL was excised, and tibial and femoral bone tunnels were drilled at the anatomical insertions using 1.8 mm Kirschner wires to maintain optimal graft isometry (Figures 3a and b). The tendon graft was passed through the tibial tunnel and then into the femoral tunnel (Figures 3c–e). The ends of the graft were secured using sutures, with knots tied up to 6 times to exceed the bone tunnel diameter, thereby mimicking an endobutton implant (Figure 3f). This knotting approach was adopted to reduce thread breakage associated with screw fixation, as previously reported by Bachy *et al*. [13] and Tian *et al*. [17]. The incision was closed in layers (arthrotomy, subcutaneous tissue, and skin) using sutures. Sterile gauze was applied as a dressing. Postoperatively, the knee was left unrestrained.

### ACL repair

The ACL repair procedure followed the same steps as the reconstruction up to arthrotomy. After exposing the joint, the femoral attachment of the ACL was transected (Figure 2a), and the ligament stump was sutured using the Krackow technique (Figure 2b). Femoral bone tunnels were created at the anatomical insertion sites using 1.8 mm Kirschner wires to ensure isometric placement of the repaired ligament. Sutures were passed through the femoral tunnels using a 23-gauge needle, then tensioned and tied in knots up to six times until they exceeded the tunnel diameter, emulating an endobutton configuration (Figure 2c). Incisions were closed in layers, and sterile gauze was applied. No immobilization was used postoperatively.

### Postoperative care and specimen collection

Following surgery, all rabbits received a 100 cc subcutaneous bolus of paracetamol, repeated every 24 h for 3 days. Cefazolin (20 mg/kg) was administered intramuscularly at 24-h intervals for 3 consecutive days. The animals were not immobilized postoperatively. Daily clinical evaluations were performed to monitor for signs of pain or complications such as wound infection, cage sores, weight loss, or dehiscence.

At 6 weeks post-surgery, rabbits were anesthetized as previously described and then euthanized with a high-dose (30–40 mL) ketamine injection. Death was confirmed by the absence of respiration, heart sounds, and reflexes. The left knee was used for biomechanical testing with fresh samples, while the right knee was processed for histological and immunohistochemical analyses.

### Histological and immunohistochemical analysis

To assess osteointegration at the tendon/liga-ment-bone interface, femur-ligament complexes (n = 3/group) were fixed in 10% neutral-buffered formalin for 24 h. Specimens were decalcified for 48 h, embedded in paraffin, sectioned longitudinally at 4 μm, and stained with hematoxylin and eosin for histological evaluation. Immunohistochemical staining was performed using the labeled streptavidin-biotin method with the Starr Trek Universal HRP Detection System from Biocare Medical, USA (catalog number 901-STUHRP700- 052623), targeting collagen types I and III.

Image acquisition was conducted using a trinocular microscope with a mounted digital camera. Images were compressed using the lossless TIFF format. The staining algorithm applied deconvolution techniques as proposed by Ruifrok *et al*. [18], followed by image segmentation for analysis.

Quantitative assessment of Sharpey’s-like fibers was conducted by two blinded pathologists, and interobserver reliability was tested through Bland–Altman analysis. Collagen fiber dimensions (longitudinal and transverse) were measured using ImageJ software (https://imagej.net/ij/) .

### Biomechanical testing

Immediately after euthanasia, femur–tibia speci-mens (n = 3/group) were prepared for biomechanical testing. All soft tissue was carefully removed, except for the graft within the joint and sutures at the tunnel exits. The femur–graft–tibia complex was secured in custom iron clamps attached to a mechanical testing apparatus. Tensile load was applied at a constant displacement rate of 10 mm/min, and both displacement and load data were recorded from the resulting load–deformation curves.

### Statistical analysis

Quantitative data are presented as mean ± stan-dard deviation (SD) for normally distributed variables and as median with range (min–max) for non-normally distributed variables. Differences between groups were analyzed using the independent t-test for normally distributed data and the Mann–Whitney U-test for non-parametric data.

## RESULTS

### Surgical outcomes

None of the rabbits in this study died during or following surgery. The administered anesthetic dosage proved effective, maintaining adequate sedation throughout the entire 30-min surgical procedure. All rabbits remained calm, and their surgical wounds healed appropriately without complications. By 1 week postoperatively, the animals resumed normal movement.

Postoperative pain was assessed using the Rabbit Grimace Scale (RbtGS), which comprises five facial action units (FAUs): Orbital tightening, cheek flattening, pointed nose, whisker changes, and alterations in ear shape and position. Each FAU is scored on a scale from 0 (absent) to 2 (obviously present). Observations were made preoperatively and 24 h after surgery. The average RbtGS score for both groups was 0 before surgery and increased to 1.3 ± 0.47 at 24 h postoperatively.

### Histological analysis of Sharpey’s-like fibers

**Figure 4:**
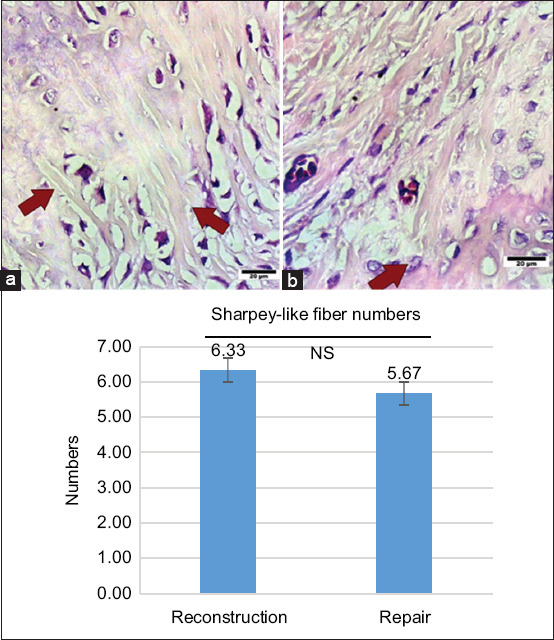
(a) Patients who underwent anterior cruciate ligament (ACL) reconstruction. Sharpey’s-like fibers are visible in more numbers than in image B (b), which is the group that underwent ACL repair.

Histological evaluation of Sharpey’s-like fibers at week 6 is presented in Figure 4. Results were independently verified by a second observer and subjected to Bland–Altman analysis, which demonstrated good interobserver agreement. The mean number of Sharpey’s-like fibers was higher in the reconstruction group (6.33 ± 0.58%) compared to the repair group (5.67 ± 1.6%). However, this difference did not reach statistical significance (p > 0.05).

### Immunohistochemical analysis of types I and III collagen

**Figure 5:**
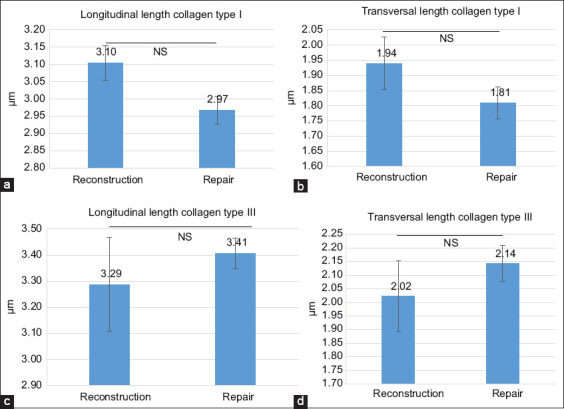
(a–d) Longitudinal and transverse fiber lengths of types I and III collagen in the reconstruction and repair groups.

The longitudinal and transverse dimensions of types I and III collagen fibers were quantitatively analyzed for both groups (Supplementary Tables 1 and 2). These findings are summarized in Figure 5. The mean longitudinal length of type I collagen fibers in the reconstruction group was 3.10 ± 0.05 μm, significantly greater than in the repair group (2.97 ± 0.04 μm; p < 0.05). Similarly, the transverse length of type I fibers was greater in the reconstruction group (1.94 ± 0.09 μm) compared to the repair group (1.81 ± 0.05 μm), though this difference was not statistically significant (p > 0.05).

**Supplementary Table 1:** Size of type III collagen fibers in the reconstruction and repair groups.

Group	Specimen	Collagen type III	

		Longitudinal length (µm)	Transverse length (µm)
Reconstruction	II.1	1.28	2.01
	II.2	1.47	2.16
	II.3	1.11	1.90
Repair	III.1	1.45	2.10
	III.2	1.34	2.11
	III.3	3.43	2.22

**Supplementary Table 2:** Size of type I collagen fibers in the reconstruction and repair groups.

Group	Specimen	Collagen type I	

		Longitudinal length (µm)	Transverse length (µm)
Reconstruction	II.1	1.15	1.89
	II.2	1.11	2.04
	II.3	3.05	1.89
Repair	III.1	2.93	1.77
	III.2	3.01	1.79
	III.3	2.96	1.87

**Figure 6:**
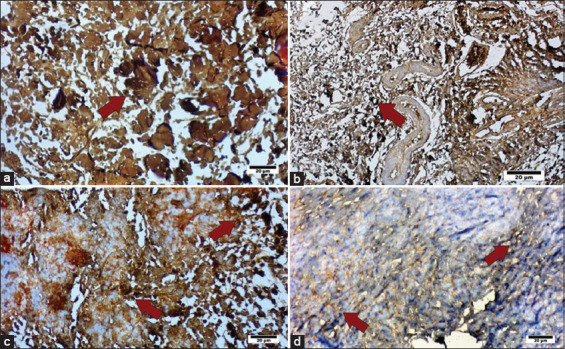
(a) The group that underwent anterior cruciate ligament (ACL) reconstruction and has a longer transverse length of type I collagen fibers than (b), which underwent ACL repair. (c) The group that underwent ACL reconstruction and has a longer transverse length of type III collagen fibers than (d) that underwent ACL repair. Bars, 20 μm.

For type III collagen, the average longitudinal lengths were 3.29 ± 0.18 μm in the reconstruction group and 3.41 ± 0.06 μm in the repair group. The transverse dimensions were 2.02 ± 0.13 μm and 2.14 ± 0.07 μm, respectively. No significant differences were observed in either dimension of type III collagen fibers between the two groups (p > 0.05). Figure 6 displays representative immunoexpression images for both collagen types in the reconstruction and repair groups.

### Biomechanical evaluation

**Figure 7:**
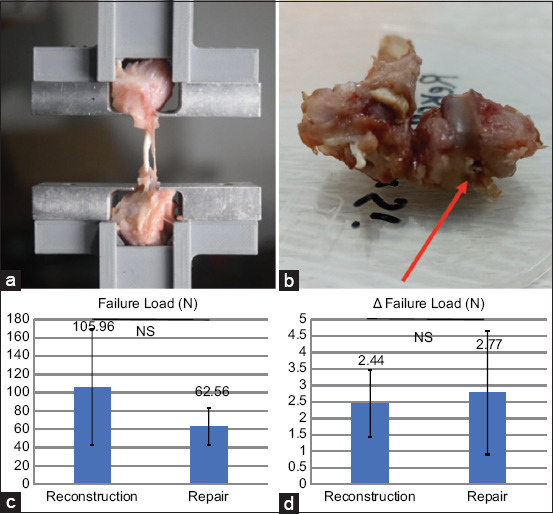
The femur-ligament-tibia complex was fixed in a custom iron device and clamped to an Instron machine for biomechanical testing. (b) The arrow indicates the site of ligament rupture after the tensile test. The location of the ligament rupture is the attachment of the anterior cruciate ligament to the femur bone, which is the bone-ligament interface. This is the location where the ligament tissue and bone meet. (c) The average failure load in the repair group was lower than that in the reconstruction group at 6 weeks postoperatively; however, the difference was not statistically significant. (d) The ligament elongation before failure (Δ L at failure load) between two groups also not statistically different.

At 6 weeks postoperatively, all specimens from both groups exhibited failure during the pull-out tensile test (Figure 7a), confirming that failure occurred at the treated bone–ligament interface (Figure 7b). The reconstruction group demonstrated a higher average load to failure (105.96 ± 63.37 N) compared to the repair group (62.56 ± 20.11 N; Figure 7c). In addition, the reconstruction group had a shorter elongation before failure (2.44 ± 1.02 mm) than the repair group (2.77 ± 1.87 mm; Figure 7d). However, these differences in failure load and elongation were not statistically significant (p > 0.05).

## DISCUSSION

Tendon and ligament-to-bone healing plays a pivotal role in determining the outcomes of ACL surgery. This healing process is inherently complex due to the anatomical and physiological challenges presented by the tendon–bone junction, where soft tissue interfaces with hard bone [17]. Ligaments are composite biological structures, primarily composed of collagen fibers embedded in an extracellular matrix, that contribute significantly to joint stability and function. Collagen is the main structural component enabling ligaments to bear mechanical loads. The mechanical properties of ligament tissue are determined by its microarchitecture and composition, predominantly comprising collagen types I and III. The relative proportions of these collagen types vary depending on the healing phase, tissue maturation, aging, and graft source. The formation of scar tissue at the tendon–bone interface generally weakens the mechanical integrity of the healing site.

Scar tissue is typically rich in type III collagen, which is more compliant than type I collagen. In contrast, type I collagen confers greater tensile strength. An experimental study involving rabbit medial collateral ligaments demonstrated that ligaments with a higher proportion of type I collagen exhibited increased stiffness compared to those with higher type III collagen content. Variations in the ratio of collagen types I and III significantly affect ligament mechanical behavior and can manifest in clinical symptoms such as impaired joint stability, restricted motion, and susceptibility to rupture [18].

Achieving effective tendon– or ligament–to–bone integration remains a major challenge in both ACL reconstruction and repair, demanding advancements in both surgical techniques and biological interventions. Unlike the union of similar tissues (e.g., bone-to-bone), the connection between ligament and bone at the enthesis is structurally and functionally complex. This study reinforces that tensile test failures consistently occur at the bone–ligament interface, underscoring this site as a biomechanical weak point in ACL repair [19].

Our findings indicate that both ACL repair and reconstruction groups demonstrated successful osteointegration at 6 weeks, as evidenced by the formation of Sharpey-like fibers extending perpendicularly from the ligament or tendon into the bone. The ACL reconstruction group exhibited a higher quantity of type I collagen fibers compared to the repair group, while type III collagen fiber dimensions did not differ significantly. Since type I collagen plays a critical role in determining ligament strength [20], its increased presence in the reconstruction group aligns with the higher average failure load observed in that group. Although there were measurable differences in failure load between the two groups, these did not reach statistical significance, likely due to variability within the data, as reflected by the high SD.

The elevated expression of type I collagen in the reconstruction group may be attributed to the broader tendon–bone contact area within the femoral tunnel, as opposed to the more limited contact area in the ACL repair model, which is confined to the proximal ligament attachment on the femur. This anatomical distinction likely influenced biomechanical outcomes, with the repair group exhibiting lower failure loads. In addition, the greater number of Sharpey’s-like fibers in the reconstruction group further supports this interpretation. These findings may help explain why ACL repair outcomes generally remain inferior to those of reconstruction, despite the theoretical benefits of the former [6, 9].

Optimally, tendon/ligament-to-bone healing should aim to recreate the native enthesis structure, comprising ligament tissue, fibrocartilage, calcified fibrocartilage, and bone – each containing type I collagen, mineral components, and small quantities of proteoglycans [17]. To overcome the inherent limitations of ACL repair, emerging strategies include biological augmentation aimed at stimulating type I collagen synthesis at the bone–ligament interface, thereby enhancing mechanical strength and structural integrity [21, 22]. Developing a reproducible ACL repair model in rabbits thus establishes a foundational platform for investigating surgical refinements and biologically enhanced repair strategies.

The tendon/ligament–bone interface is exposed to heterogeneous mechanical environments, which further complicate integration due to the transition from soft connective tissue to hard bone. ACL repair is typically indicated for proximal ACL tears (Sherman types I and II) with good tissue quality, especially for femoral avulsion injuries. [10]. However, the current arthroscopic techniques remain technically challenging for mid-substance ACL ruptures. For this reason, our study utilized a Sherman type I rupture model, involving a tear near the femoral attachment.

## CONCLUSION

This study successfully established a novel and reproducible ACL repair model using New Zealand White rabbits, enabling a direct comparison with ACL reconstruction in terms of osteointegration, collagen morphology, and biomechanical performance. At 6 weeks postoperatively, both the repair and reconstruction groups exhibited signs of tendon/ligament-to-bone integration, as evidenced by the presence of Sharpey’s-like fibers. However, the ACL reconstruction group demonstrated superior biomechanical strength, characterized by a higher average tensile failure load (105.96 ± 63.37 N vs. 62.56 ± 20.11 N), and a significantly greater longitudinal length of type I collagen fibers, a key determinant of ligament strength. Although type III collagen fiber dimensions did not differ significantly, the structural advantage conferred by enhanced type I collagen expression in the reconstruction group underscores its mechanical superiority.

One of the key strengths of this study lies in the successful implementation of a cost-effective, small-animal model for ACL repair, which offers practical advantages for histological, biomechanical, and translational research. In addition, the comparative evaluation of collagen morphology and Sharpey’s-like fiber formation provides valuable insights into the biological underpinnings of tendon/ligament-to-bone healing.

However, several limitations should be acknowledged. First, the small sample size and single time-point analysis limit the statistical power and preclude longitudinal assessment of the healing and remodeling processes. Second, post-operative mechanical loading was not controlled, which may have introduced variability in the healing response. Third, while the rabbit model provides a feasible and scalable platform, it may not fully replicate the biomechanical and physiological complexity of the human knee joint.

Future studies should explore multi-timepoint evaluations to capture the dynamic phases of osteointegration, incorporate controlled mechanical loading conditions, and investigate the effects of biological augmentation – such as stem cell therapy, growth factors, and scaffold-based delivery systems – on enhancing ACL repair. Such efforts will be instrumental in bridging the translational gap between experimental models and clinical applications, ultimately contributing to improved outcomes in ligament repair surgery.

## AUTHORS’ CONTRIBUTIONS

AY: Conceptualized the study, methodology, investigation, data curation, and drafted the manuscript., AIM: Investigation, data curation, formal analysis, validation, and supervised the study. NNH: Investigation, supervised the study, and reviewed and edited the manuscript. HA: Investigation, data curation, formal analysis, validation, and supervised the study. NCB: Conceptualized and supervised the study, validation, and reviewed and edited the manuscript. NA: Conceptualized and supervised the study, methodology, data curation, investigation, validation, and reviewed and edited the manuscript. All authors have read and approved the final version of the manuscript.
